# RNA-QC-chain: comprehensive and fast quality control for RNA-Seq data

**DOI:** 10.1186/s12864-018-4503-6

**Published:** 2018-02-14

**Authors:** Qian Zhou, Xiaoquan Su, Gongchao Jing, Songlin Chen, Kang Ning

**Affiliations:** 1 Yellow Sea Fisheries Research Institute, Chinese Academy of Fishery Sciences, Key Laboratory for Sustainable Development of Marine Fisheries, Ministry of Agriculture, and Laboratory for Marine Fisheries Science and Food Production Processes, Qingdao National Laboratory for Marine Science and Technology, Qingdao, Shandong 266071 China; 2 Shandong Key Laboratory of Energy Genetics, CAS Key Laboratory of Biofuels, and Single Cell Center, Qingdao Institute of Bioenergy and Bioprocess Technology, Chinese Academy of Sciences, Qingdao, Shandong 266101 China; 3 University of Chinese Academy of Sciences, Beijing, 100049 China; 4 Department of Bioinformatics and Systems Biology, Key Laboratory of Molecular Biophysics of the Ministry of Education, Hubei Key Laboratory of Bioinformatics and Molecular Imaging, College of Life Science and Technology, Huazhong University of Science and Technology, Wuhan, Hubei 430074 China

**Keywords:** Quality control, RNA-Seq, Contamination identification, Alignment statistics, Parallel computing

## Abstract

**Background:**

RNA-Seq has become one of the most widely used applications based on next-generation sequencing technology. However, raw RNA-Seq data may have quality issues, which can significantly distort analytical results and lead to erroneous conclusions. Therefore, the raw data must be subjected to vigorous quality control (QC) procedures before downstream analysis. Currently, an accurate and complete QC of RNA-Seq data requires of a suite of different QC tools used consecutively, which is inefficient in terms of usability, running time, file usage, and interpretability of the results.

**Results:**

We developed a comprehensive, fast and easy-to-use QC pipeline for RNA-Seq data, RNA-QC-Chain, which involves three steps: (1) sequencing-quality assessment and trimming; (2) internal (ribosomal RNAs) and external (reads from foreign species) contamination filtering; (3) alignment statistics reporting (such as read number, alignment coverage, sequencing depth and pair-end read mapping information). This package was developed based on our previously reported tool for general QC of next-generation sequencing (NGS) data called QC-Chain, with extensions specifically designed for RNA-Seq data. It has several features that are not available yet in other QC tools for RNA-Seq data, such as RNA sequence trimming, automatic rRNA detection and automatic contaminating species identification. The three QC steps can run either sequentially or independently, enabling RNA-QC-Chain as a comprehensive package with high flexibility and usability. Moreover, parallel computing and optimizations are embedded in most of the QC procedures, providing a superior efficiency. The performance of RNA-QC-Chain has been evaluated with different types of datasets, including an in-house sequencing data, a semi-simulated data, and two real datasets downloaded from public database. Comparisons of RNA-QC-Chain with other QC tools have manifested its superiorities in both function versatility and processing speed.

**Conclusions:**

We present here a tool, RNA-QC-Chain, which can be used to comprehensively resolve the quality control processes of RNA-Seq data effectively and efficiently.

## Background

RNA-Seq has become a routinely and extensively applied approach for transcriptome profiling that relies on high-throughput sequencing (HTS) technologies, which provides a far more profound and precise measurement at the transcript level than microarray and other traditional gene expression analysis methods [1]. It could also be used for identification of novel transcripts [2], alternative spliced variants [3] and gene fusion events [4]. However, due to intrinsic limitations of HTS technologies and RNA-Seq protocols, quality problems are quite common in raw RNA-Seq data. In addition to “HTS-common” quality problems that are generally present in all kinds of HTS data, such as sequencing-quality of raw read and contamination from other species [5], there are some “RNA-Seq-specific” quality issues, such as ribosomal RNA (rRNA) residual, RNA degradation and varied read coverage. Therefore, before downstream analysis, raw RNA-Seq data must be checked and processed by quality control (QC) procedures to ensure accurate transcript measurements and correct knowledge acquirements from the data.

Currently, a number of tools [6] are available for HTS data QC, such as FastQC (http://www.bioinformatics.babraham.ac.uk/projects/fastqc/), FASTX-Toolkit (http://hannonlab.cshl.edu/fastx_toolkit/), QC-Chain [5], NGS QC Toolkit [7]. However, most of them mainly focus on trimming of general HTS data, but not for specific RNA-Seq QC problems. Though some tools are designed specifically for RNA-Seq data, they suffer from different kinds of restrictions. For instance, RSeQC mainly provides QC summary statistics of read alignment and relies on UCSC (the University of California, Santa Cruz) Genome Browser (http://genome.ucsc.edu/) to some extent [8]. RNA-SeQC can evaluate different quality aspects of reads and alignments [9]. However, both of them lack functions of sequence trimming and contamination filtering, and run slowly. Therefore, there is a pressing need for a new and powerful QC method for RNA-Seq data.

Here we present RNA-QC-Chain, an easy-to-use, highly efficient and one-stop QC tool for RNA-Seq data. With both quality check and data processing capability, RNA-QC-Chain includes three related functional components, called Parallel-QC, rRNA-filter and SAM-stats. In addition to covering most types of quality assessments offered by currently available tools, RNA-QC-Chain can filter out the poor-quality reads and contaminations, and generate the ready-to-use data for downstream analysis. Notably, parallel computation is embedded in RNA-QC-Chain, which could significantly accelerate its processing speed and makes it an extremely fast QC software.

### Implementation

#### The workflow of RNA-QC-chain

**Fig. 1:**
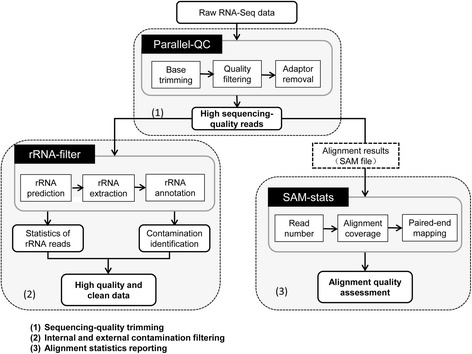
The workflow and functions of RNA-QC-Chain. Firstly, reads with sequencing quality defects are trimmed by Parallel-QC, secondly, internal (ribosomal RNA) and external (non-target species) contaminations are identified and filtered by a tool called rRNA-filter. Finally, multiple statistics based on the alignment results are reported by a tool called SAM-stats

RNA-QC-Chain has three sequential QC procedures, with parallel computation as the backbone to provide a complete and high-performance QC solution for RNA-Seq data (Fig. 1). Firstly, Parallel-QC [5] is used to assess and trim low sequencing-quality reads. Secondly, by a module called rRNA-filter, rRNA fragments are identified, extracted and used to identify the contaminating species. Finally, based on results of reads alignment (to reference genome), multiple mapping metrics are provided to evaluate the RNA-Seq data and experiment by another embedded module called SAM-stats.

### Sequencing-quality assessment and trimming

Parallel-QC has been introduced in our previously established QC method QC-Chain, which is suitable for all kinds of NGS raw data and can accomplish basic read processing procedures of base trimming, read trimming and adapter trimming. Base trimming cuts bases with quality value lower than Q (default Q value is 20) at each end of reads. Read trimming filters reads by an given parameter-pair (Q, R%): reads containing more than R% (default R value is 10) of bases with quality value lower than Q will be trimmed. Adapter trimming identifies and drops reads with sequencing adapter or PCR primer sequences, using either a list of ready-to-use Illumina standard tag sequence or user-specified adaptor sequences as a reference. Pairing information could be kept in every processing procedure, which is essential to many subsequent transcript/RNA analyses. Notably, the duplications in RNA-Seq data should not be removed because this information is closely relative to RNA abundance calculation. In our tests, parameters for quality trimming was 20, 10% and the default list of tag sequences were used in adapter trimming.

### Internal and external contamination filtering

Ribosomal RNA was considered as the internal contaminations since they are from the target sequencing species, while external contaminations were defined as reads from foreign species other than the sequencing targets. In RNA-QC-Chain, a tool called “rRNA-filter” was developed to extract rRNA reads and to identify both internal and external contaminations. Firstly, the rRNA fragments were predicted from input sequences using HMM (Hidden Markov Model) search of HMMER [10] and then extracted out. The rRNA pattern model was constructed by all 16S/18S/23S/28S rRNA fragments from SILVA database (version 123) [11]. Since the HMM algorithm does not rely on the annotation of the source genome of the rRNA, but the pattern of the rRNA sequences, the RNA-QC-Chain makes the removal of the rRNA fragment to be alignment and annotation free. As a result, internal contaminations including both small (16S/18S) and large subunit (23S/28S) rRNA sequences were removed. Then, the extracted 16S or 18S rRNA fragments were mapped onto the rRNA databases of SILVA for prokaryotic and eukaryotic identification, respectively [12]. Based on the assignment of the classification terms, the taxonomical components of the RNA-Seq data were produced, which indicated whether there was contaminating species, and if so, what these species was.

### Alignment statistics reporting

Furthermore, using a script called “SAM-stats”, RNA-QC-Chain provides the assessment profiles based on read alignment. These assessments include multiple aspects:

1. Reads:

- Number of total reads and mapped reads;
- Number of reads mapped to each specific genomic region (such as CDS and exon), which is defined in the user-specified gene model (GTF or GFF) file;
- Number of reads mapped outside the genomic regions specified in the gene model (GFF/GTF) file.

(2) Coverage (gene is called “expressed” when 50% of its sequence are mapped by reads):

- Number of expressed gene and its proportion out of all genes;
- Coverage of each gene and the overall coverage distribution;
- Distribution of mapped reads.

(3) Mapping:

- Genebody coverage bias: average mapping coverage of each base position over the genes (scale all of the transcripts into 100 bp windows);
- Strand specificity: reads mapped to positive/negative strands, respectively;
- Library complexity: number of reads with varied mapping starting point.

(4) Pair-ended read mapping:

- Number of paired mapped reads;
- Number of discordantly mapped pairs;
- Insert size distribution of mapped read pairs.

### Input and output

Within the RNA-QC-Chain software package, sequencing-quality trimming and contamination filtering steps could be performed based on sequencing data in either FASTA or FASTQ format. Alignment statistics reporting step accepts alignment results in SAM format and gene structure file in GTF or GFF format.

Output formats depend on the specific steps of RNA-QC-Chain. Sequencing-quality trimming step exported filtered high-quality read file, trimmed read file and an analysis report. Contamination filtering step exported the extracted rRNA which were decomposed into sub data files with rRNA classification and filtered rRNA-free data. An active graph and text report of the taxonomy information were also generated to indicate the possible contaminating species. Alignment statistics reporting step exported a general analysis report, showing data statistics and gene mapping information. In addition, four figures, along with a detailed statistical text file for each plot, were also generated, including mapping region distribution, coverage distribution, genebody coverage bias and insert size distribution of mapped read-pairs.

### Parallel computing and optimizations

Parallel computation optimization was applied on RNA-QC-Chain in sequencing-quality trimming and contamination filtering steps. Input reads were loaded to RAM and then distributed to multiple CPU cores to be processed in parallel. Number of threads could either be automatically allocated based on the hardware configuration, or be assigned by users. The task loading balance was optimized based on the dynamic scheduling technique using C/C+ OpenMP library to maximally take the advantage of computing resources. In addition, since the sequence QC is also data-intensive, in each of the three QC steps, most of the data exchange among different steps could be finished in RAM so that all steps were conducted with only one disk I/O operation.

### Datasets used for testing

We used four datasets to test the performance of RNA-QC-Chain (Table 1). Dataset 1 was a real in-house sequenced RNA-Seq data of algae species Nannochloropsis. The shotgun paired-end (PE) cDNA library with an insert size of 280 bp was constructed and then sequenced by Illumina Hiseq2000 sequencing platform, producing a total of 7,045,705 read pairs with a read length of 100 bp at each end (SRA accession number SPR032930). Dataset 2 was a semi-simulated data, integrating a real RNA-Seq data for a Sprague–Dawley rat sample, and simulated contaminating reads from yeast *Saccharomyces cerevisiae*. The rat sequences were downloaded from National Center for Biotechnology Information (NCBI) (SRA ID: SRX871031, SRR1795728), with a read length of 51 bp. The simulated yeast sequences were generated from genome of *S. cerevisiae* S288C using DWGSIM (https://github.com/nh13/dwgsim) with a genome coverage of 200 and a same read length of 51 bp as that of rat. Finally, the merged dataset has a total of 18,340,356 reads and the data size was 935 Mbp. Dataset 3 and Dataset 4 were human RNA-Seq data, which were produced under the ENCODE project and downloaded from Gene Expression Omnibus database (GEO accession number GSM958728) with data sizes of 9.6 Gb and 16.4 Gb, respectively.

**Table 1:** Summary of datasets used in this paper

Name	Type	Species	No. of read	Read length (bp)	Size (Gb)
Dataset 1	real data	microalgae (*Nannochloropsis oceanica*)	7,045,705	2 × 100	1.4
Dataset 2	semi-simulated data	Real: Sprague–Dawley rats (*Rattus norvegicus*)	9,809,056	51	0.9
		Simulated: yeast *(Saccharomyces cerevisiae)*	8,531,300	51	0.4
Dataset 3	real-data	Human (*Homo sapiens*)	25,536,632	2 × 75	9.6
Dataset 4	real-data	Human (*Homo sapiens*)	54,477,454	2 × 75	16.4

All data analysis were performed on one single rack server with dual Intel Xeon X5645 CPU (12 cores and 24 threads in total), 72 GB RAM.

## Results and discussion

Compared to traditional technology like microarray, RNA-Seq has a higher productivity and better resolution, therefore, it has become the mainstream of high throughput and large scale RNA-level study. However, sequencing errors and contaminations may be introduced into the raw data during the library preparation, sequencing and base calling steps. Therefore, quality control is a first essential step in bioinformatics analysis of RNA-Seq data. RNA-Seq measures the abundance and structure of genes at the RNA level, and employs different analytical approaches compared with those for DNA-Seq data: firstly, DNA is quite stable and the DNA sequences are highly constant, while RNA are fragile and gene expression values are very dynamic; secondly, all DNA sequences could be recovered when the sequencing depth is high enough, while sequencing bias may occur to a higher level in RNA-Seq data. These different features place distinguished and high demands for accurate QC on RNA-Seq data to ensure highly reliable subsequent analytical results. RNA-QC-Chain was developed based on our published QC tool called QC-Chain, which provides basic QC solutions for general HTS data. They share similar functionalities of sequencing quality trimming [5] and rRNA identification. However, RNA-QC-Chain can identify all kinds of rRNA reads in SILVA database and automatically remove them, including 16S, 23S, 18S and 28S rRNA, while QC-Chain can only identify 16S or 18S rRNA and cannot remove the identified rRNA reads. Another difference is that RNA-QC-Chain can perform the alignment statistics, which is not applicable in QC-Chain. Therefore, RNA-QC-Chain has essential functional extensions that are particular for RNA-Seq data.

### Sequencing quality trimming

**Fig. 2:**
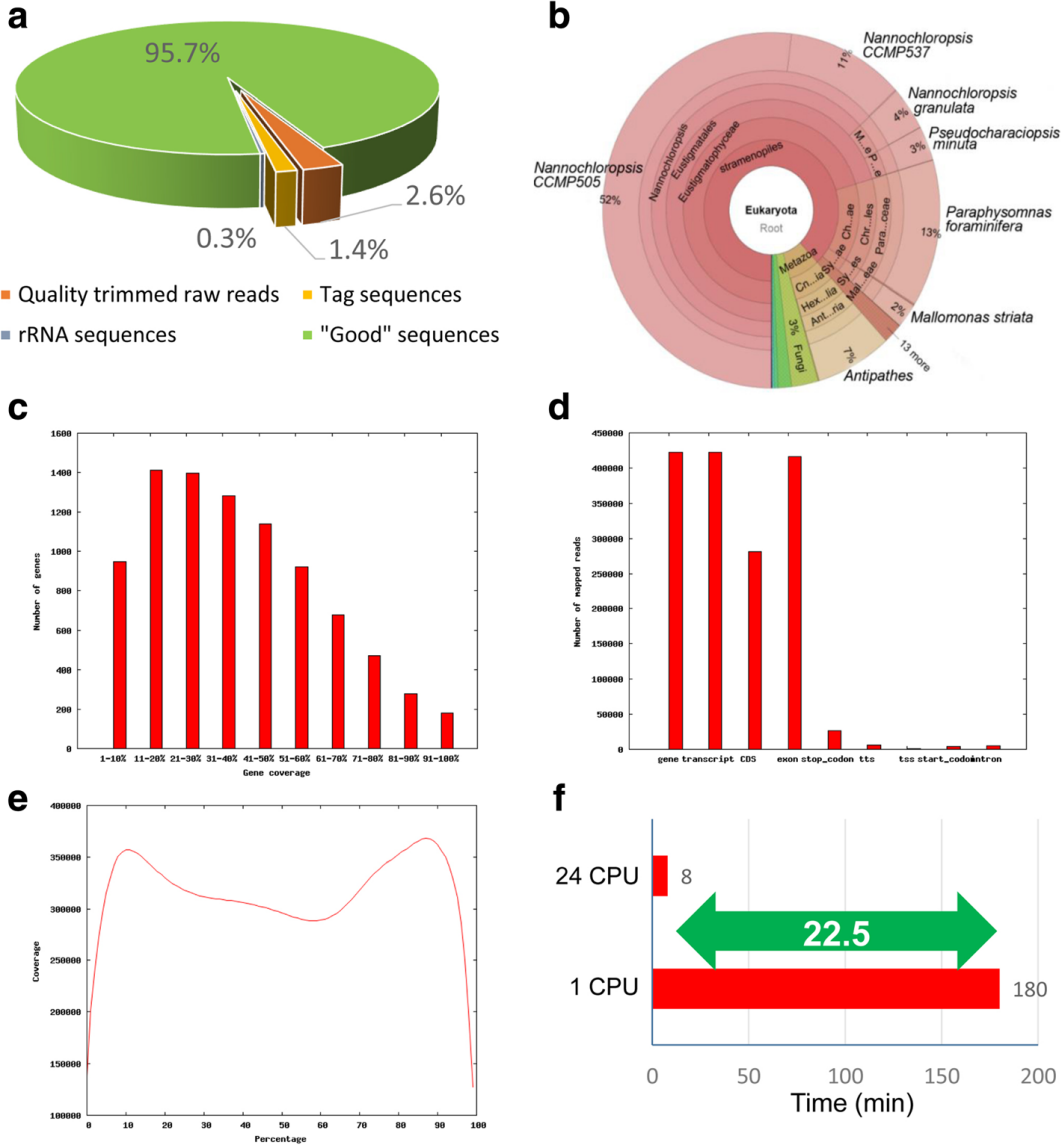
Selected outputs of RNA-QC-Chain for a RNA-Seq data of Nannochloropsis (Dataset 1). **a** Sequencing-quality and rRNA measurements. **b** External contamination screening by 18S rRNA identification. **c** Distribution of read coverage on gene. **d** Distribution of mapped reads over different genomic region. **e** Distribution of read coverage over genebody. **f** Comparison of running time of parallel and serial computation (speed-up shown in arrow)

Sequencing quality trimming of raw reads is absolutely necessary for all kinds of HTS data. Therefore, although there are a number of tools that can perform this step, we integrated Parallel-QC in our RNA-QC-Chain, to make our pipeline as a one-stop and convenient tool for RNA-Seq data QC. Common quality issues for raw reads include base quality, tag sequences, GC content and duplications. Notably, the GC content varies in different species and regions within genomes. Therefore, although it is difficult to identify the precise contaminating species using the GC content, thus shift of GC content or multiple GC peaks could be used to indicate whether there is contaminations in this data. For Dataset 1, 20.65% of the raw reads were removed by sequencing quality trimming step with default parameters and 8.01% of the raw reads were trimmed as tag sequences (Fig. 2a).

### Contamination screening and filtration

NGS data often suffers from contaminations, which may extensively influence data yield and analytical result. It is an essential QC procedure to determine whether the data is contaminated, what the contaminations are and then filter out the contaminations. For RNA-Seq data, the contaminations can be classified into two types of “internal” (rRNA reads) and “external” (reads from foreign species) according to their source.

In extracted total RNA, up to 80–90% are rRNA sequences, thus a high quality RNA-Seq experiment requires an intact total RNA extraction and efficient rRNA removal. However, by current technologies in the sample preparation stage, rRNA cannot be easily and fully digested [13]. Therefore, a high-performance rRNA removal operation is needed in the data QC stage. Moreover, it is difficult to estimate whether there are external contaminations by “wet” experiment before the sequencing data is produced, but we can measure them by bioinformatics analysis. Some standalone applications can be used to identify contaminations. For example, SortMeRNA can identify rRNA reads against SILVA rRNA database [14]. However, it cannot report the taxonomic assignment thus is unable to indicate what contaminations are. FastQ Screen (https://www.bioinformatics.babraham.ac.uk/projects/fastq_screen/) and MGA [15] are also commonly used for contamination screening. However, due to their dependency on the reference genomes, they cannot complete unknown contamination identification. After that, we developed a tool named rRNA-filter to identify and extract rRNA reads, followed by a detection of all organism species involved in the data by rRNA annotation. For Dataset 1, a total of 21,630 rRNA reads (0.25% of the raw data) were predicted and extracted (Fig. 2a). Among them, 985, 8293, 2985 and 9367 reads were identified as 16S, 18S, 23S and 28S rRNA sequences, respectively. Then, the identified rRNA reads were used to examine possible external contaminations. From the taxonomy graph obtained based on 18S rRNA alignment results, we observed that the dominant eukaryotic species was Nannochloropsis, which was our target sequencing species (Fig. 2b). In addition, a high diversity but no dominant bacteria species was identified by 16S rRNA classification, which probably resulted from random read alignment. Therefore, we would conclude that there was no significant contaminating reads included in this sample. Sometimes a small fraction of reads may map to phylogenetically close species due to random alignment. In such cases, people need to draw a conclusion according to combined consideration of the reault of rRNA-filter, the fraction of alignment to each species, background knowledge of the sample and clues from other analysis, such as GC content.

**Fig. 3:**
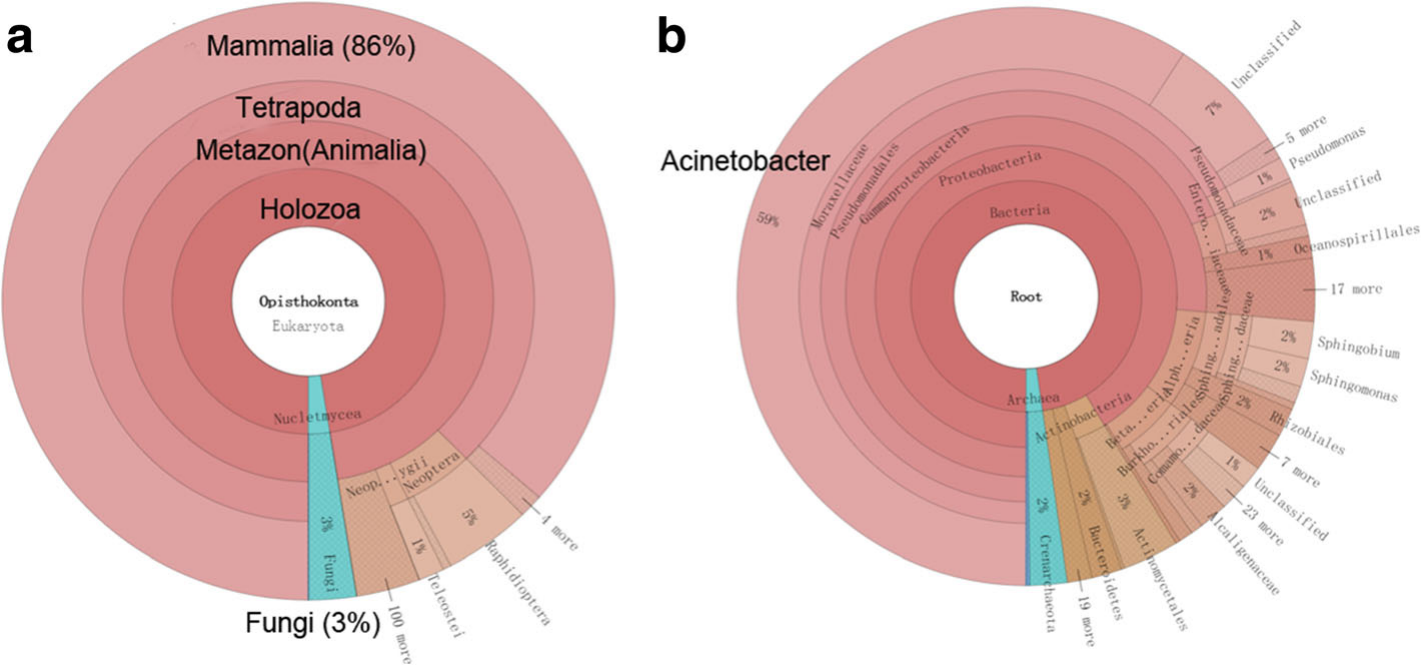
Contamination identification for a semi-simulated RNA-Seq data (Dataset 2) using RNA-QC-Chain. **a** Eukaryotic species identified by 18S rRNA screening. **b** Prokaryotic species identified by 16S rRNA screening

In Dataset 2, we artificially added some yeast data as external contaminations but are not aware in advance that bacteria reads were also included in the downloaded rat RNA-Seq data. As a matter of fact, these reads significantly reduced the sequencing depth of the rat transcripts. The identification of these contaminating species can help imply in which sample preparation or library construction step it might be introduced into the sample, and thus give instructions to avoid similar accident. Firstly, we directly aligned the reads to the reference genome of SD rat (UCSC rn5). The overall alignment rate was only 34.4%, which was significantly lower than that the theoretical proportion of the rat reads in Dataset 2 (53.5%). Then we checked the read quality using Parallel-QC, and only 43.7% of the total reads were retained as “good reads”, indicating that more than half of the reads had poor sequencing quality. After that, we used rRNA_filter to identify what contaminations may be in the data. The number of identified 18S was 74,832 and 18S rRNA survey detected reads from both rat and fungi (Fig. 3a), which is consistent to the designed eukaryotic species constitution of Dataset 2. Simultaneously, a total of 9303 16S rRNA were identified and species survey suggested that reads from bacteria might also be contained in this sample (Fig. 3b). Therefore, there were two kinds of quality issues of the Dataset 2: poor sequencing quality and bacteria contaminations. To check the correctness, we further investigated the unmapped reads using BLAST to NT database, and results showed that a number of reads aligned to bacteria. Therefore, even when the RNA-Seq data has a single end and a very short read length of 51 bp (Dataset 2), both the eukaryotic and prokaryotic contaminations can be successfully detected, demonstrating a high accuracy and efficiency of contamination identification of rRNA-filter.

On the other hand, our results demonstrated that the sequencing quality trimming and contamination identification steps are absolutely necessary and important for the QC of RNA-Seq data, because either low sequencing quality reads or contaminations may result in poor usable data yield and thus might damage further downstream analysis results.

Herein we should point out that RNA-QC-Chain has a limitation in external contamination identification in very rare cases when rRNA sequences are not involved in the data, since the foreign organism identification is based on rRNA annotation.

### Alignment statistics metrics

Read alignment to reference genome is essential for most downstream analysis of RNA-Seq data. Quality check on the read alignment results can indicate how well the target RNA was captured, amplified and sequenced, thus provides a comprehensive insight into the quality of RNA-Seq experiment and data. Some quality problems, such as incomplete mRNA amplification, sequencing bias and inconsistent coverage, cannot be detected by the quality trimming on raw reads and contamination filtering steps, but can be effectively presented by statistics on alignment result. Therefore, quality assessment of read alignment has caused great concerns [16].

We took Dataset 1 as an example to manifest the performance of the alignment statistics reporting step of RNA-QC-Chain. Generally, 85.8% (8940) of the total Nannochloropsis genes (10414) expressed in this sample. Furthermore, detailed plots of “gene coverage”, “read distribution” and “genebody coverage bias” implied mRNA integrity and sequencing bias in this sample. Specifically, most of the expressed genes were mapped at 10–50% coverage (Fig. 2c). The overall read distribution plot showed that 1,417,677 reads mapped to exons, while much fewer reads (5205) mapped to introns (Fig. 2d). Figure 2e illustrated the global coverage over the genebody, inferring there was slight alignment bias along the genes in this sample. This parameter can help indicate whether the transcripts were completely transcribed, and a significantly low coverage at 3′ and 5′ ends may cause difficulty in accurately recognizing the start and stop sites of transcripts for downstream analytical tools. Due to the innate features of RNA-Seq data, such as alternative splicing and different expression patterns, the indexes listed above may vary in different RNA-Seq samples, and these assessment can provide a global indication of the data status. We also observed that the insert distance of mapped read pairs was around 250 bp in the insert size distribution plot, which was consistent to the designed insert size of the PE library. The “strand specificity” monitors the template strand used in the sequencing. Theoretically, a mapping ratio of 99%/1% and 50%/50% is reasonable for strand-specific and non-strand-specific libraries, respectively. The library size was 158,216 for Dataset 1, which measured the diversity of mapping sites and reflected whether the RNA was randomly fragmented. In most cases, a high read diversity is expected and will benefit further analysis. In addition, detail mapping information for each gene, including number of mapped reads, gene coverage and read depth were reported in a file called “Gene_report.txt”.

### User friendliness and flexibility

RNA-QC-Chain is an easy-to-use and flexible tool. The entire QC process can be completed with simple inputs. Neither third-party files nor reference rRNA files are needed, as these required data has been integrated in the software. Additionally, each of the three processing steps can be completed using a single command line and the relatively independent three steps provide a flexible analytical strategy. Users can choose each step according to their needs and the result of each step can get mutually support. For example, both the identification of bacteria in the second step and the significant low alignment rate reported in the third step indicated and mutually confirmed that there was contaminations in Dataset 2.

### Parallel computing

By RNA-QC-Chain, all analytical tasks are automatically distributed to different threads with dynamic scheduling for optimization of the computing loading balance, and the shared memory space among all threads also significantly reduced the RAM usage. Compared to other pipelines that integrated with multiple software packages to depend on additional I/O operations for data transfer, the high efficiency I/O strategy of RNA-QC-Chain significantly decreased the entire analytical time. Consequently, RNA-QC-Chain has the ability of fast processing massive scale of RNA-Seq data. For Dataset 1, the whole QC process was completed within 8 min using 24 CPU threads, whereas serial computation cost more than 180 min (22.5 folds of time cost) with the same hardware and environmental system configurations (Fig. 2f).

### Comparisons with other QC tools for RNA-Seq data

We compared RNA-QC-Chain with two other QC tools for RNA-Seq data, RNA-SeQC and RSeQC, which were developed by different techniques with different features, and have been widely used for RNA-Seq quality checking [17]. RNA-SeQC is implemented in Java, which is installation free [9]. RSeQC is a toolkit package that is consisted of a number of python scripts [8]. RNA-QC-Chain is developed based on C++. Detailed comparisons of the three QC tools are listed in Table 2**.**

**Table 2:** Comparison of functions and features of different QC tools for RNA-Seq data

	RNA-QC-Chain	RSeQC	RNA-SeQC
Functions			
Quality evaluation of raw reads	Yes	Yes	No
Quality trimming of raw reads	Yes	No	No
rRNA detection	Yes	Yes	Yes
rRNA removal	Yes	No	No
Contaminating species identification	Yes	No	No
Alignment statistics	Yes	Yes	Yes
Features			
Language	C++	Python and C	Java
Input file (except commonly required files)	None	A chromosome size file and a BED file	An indexed bam file, a reference sequence, the index of reference sequence and a sequence dictionary
Output format	FASTQ/FASTA, TXT, PNG, HTML	PDF, TXT	HTML
Usage	One command line for each step	Multiple separate scripts	One command line
rRNA reference file	A built-in rRNA database	Requires user to provide	Requires user to provide
Visualization dependence	Gnuplot	UCSC Genome Browser or R scripts	N/A
Parallel computation	Yes	No	No
Running time for Dataset 1 (min)	8	55	120

Firstly, in functional aspects, both RSeQC and RNA-QC-Chain can evaluate the sequencing quality of raw reads, but only RNA-QC-Chain can complete the trimming of poor quality sequences and produce the trimmed reads. For contamination filtering, all the three tools can estimate how many reads are possibly originated from rRNA genes, however, RNA-QC-Chain is the only one that is capable of automatically removing them, while other tools needs the user to provide an rRNA reference file. In addition, neither RNA-SeQC nor RSeQC can identify the contaminating foreign species in the data, whereas RNA-QC-Chain is unique in this function. Therefore, when using RNA-SeQC and RSeQC, users have to turn to other data processing tools to filter the poor-quality reads and contaminations, but RNA-QC-Chain can directly produce the usable data for further analysis. For the statistical metrics on alignment result, all the three methods can provide some important measurements, such as mapped read count, strand specificity, read distribution, 3′/5′ bias and coverage. RNA-SeQC has a specific module to estimate the expression correlation of multiple samples [9]. RSeQC can check the sequencing saturation status [8]. These measurements are based on reads per kilobases per millionreads (RPKM) values, which are not necessary for all kinds of RNA-Seq analysis. Therefore, we did not involve measurements based RPKM values in RNA-QC-Chain.

Secondly, the usage of the three tools is different. RNA-SeQC completes the alignment assessment with a single command line, which is convenient but may be time-consuming when not all the QC aspects are required. RSeQC is consisted of a number of python scripts, with each script for a specific assessment. Therefore, it is time-economic to assess a certain quality aspect, but the operation will be very complex when running all the scripts. Giving considerations to convenience and flexibility, RNA-QC-Chain contains three relatively independent QC components, each with a single command line, to perform both quality check and data processing functions.

Thirdly, the input and output files are quite different between the three tools. For input files, a data file, a gene structure file and a read alignment file are commonly required for all the three tools. For RNA-QC-Chain, no additional files are needed. In contrast, both RNA-SeQC and RSeQC have other particular input requirements. RNA-SeQC requires a reference sequence and a sequence dictionary. Some special functional modules even need more additional files [9]. To use RSeQC, a chromosome size file should be downloaded from UCSC Genome Browser and a BED file of the sequencing species need to be downloaded from UCSC or converted from GTF/GFF format file [8]. Therefore, it would be more suitable for organisms with reference genome and annotations listing in UCSC Genome Browser. In particular, for both RNA-SeQC and RSeQC, users have to prepare a reference rRNA sequence file for rRNA reads detection. On the contrary, RNA-QC-Chain applied a different strategy with a built-in comprehensive rRNA database, making the prediction of rRNA reads more precise, comprehensive and easy-to-operate.

For output, RNA-SeQC can export HTML reports containing mainly tables. The output of RSeQC are multiple plots and texts, and the result visualization relies on R scripts. The output of RNA-QC-Chain demonstrates different quality aspects of data in different formats, including the quality-filtered sequence file in FASTA/FASTQ format that can be directly used for downstream analysis, rRNA reads filtered out, an active graph in HTML report suggesting the contamination information and plots/texts for the alignment metrics.

**Fig. 4:**
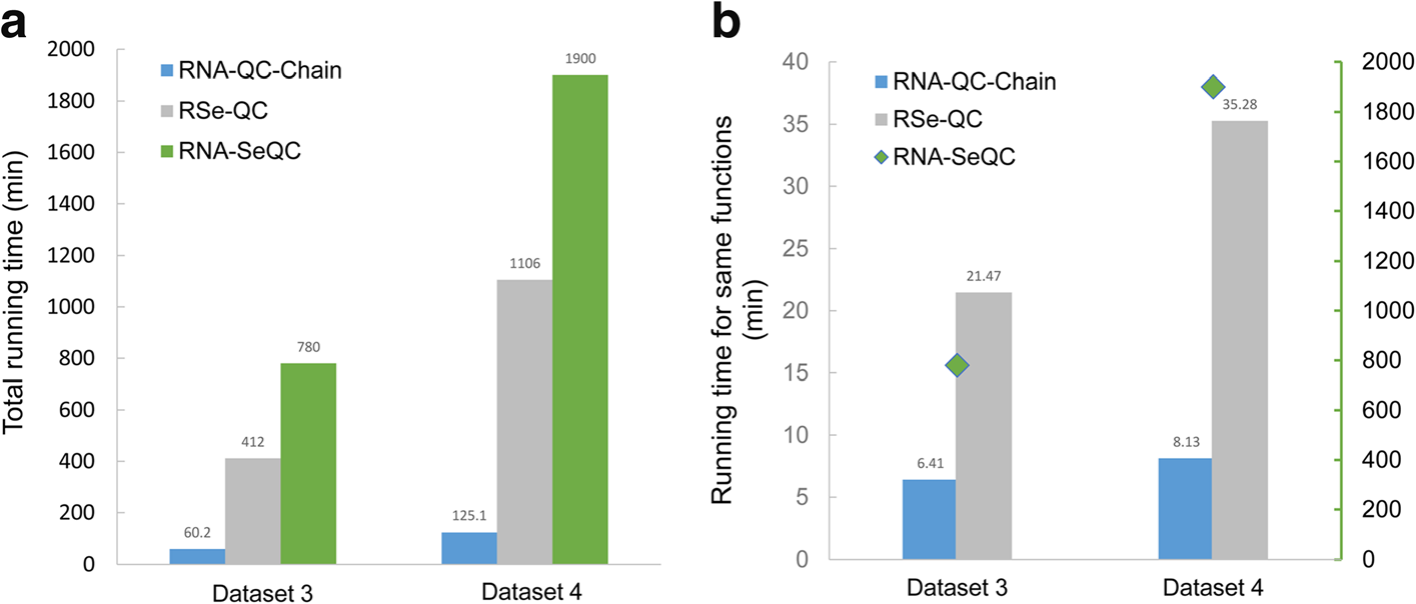
The running time of RNA-QC-Chain compared to RSeQC and RNA-SeQC using testing datasets. **a** The total running time of the complete functions of each tool. **b** The running time of the same functions of the three tools. The used scripts of RSeQC in this test included bam_stat.py, geneBody_coverage.py, infer_experiment.py, inner_distance.py, read_duplication.py, read_GC.py, read_distribution.py and split_bam.py

Finally, HTS data analysis is both data- and computation-intensive, therefore, there is a thirsty requirement for high performance computation. RNA-QC-Chain was developed to cope with such need for fast analyses. The running time of RNA-QC-Chain, RSeQC and RNA-SeQC was compared using datasets with different data size. Benefited by the whole-process parallel scheduling, multi-thread memory sharing and C++ programming, RNA-QC-Chain achieved approximately 7–13 times faster than other tools (Fig. 4a). Since many of the outputs are not commonly available among the three tools, we further selected the same/similar functions of the three tools and compared their running time. The used scripts of RSeQC included bam_stat.py, geneBody_coverage.py, infer_experiment.py, inner_distance.py, read_duplication.py, read_GC.py, read_distribution.py and split_bam.py. For example, for dataset 3 RNA-QC-Chain only took about 6 min, while RSeQC and RNA-SeQC ran more than 20 and 700 min, respectively (Fig. 4b). This high speed running demonstrated the capability of RNA-QC-Chain to accomplish the analysis of data in huge size and large amount of samples, for which is essentially important for high efficient bioinformatics analysis.

## Conclusions

RNA-QC-Chain provides a comprehensive, one-stop and high efficient solution for RNA-Seq data QC, which would be very beneficial for knowledge discovery from RNA-Seq data. It performs sequencing quality trimming for raw read, automatic contamination identification and filtering, and alignments statistics reporting, and is able to produce data ready for further downstream analysis. It is very user-friendly with convenient and flexible usage. Comparisons with other QC tools indicated that RNA-QC-Chain outperformed in both function and speed. Parallel computation makes its running significantly faster than serial computation and other QC tools. This tool can be used as the QC tool for the first step in analysis pipeline of RNA-Seq data to quickly provide the data quality information and the filtered reads ready for downstream analysis.

## Availability and requirements

**Project name:** RNA-QC-Chain

**Project home page:** http://bioinfo.single-cell.cn/rna-qc-chain.html or http://124.16.150.212/rna-qc-chain.html

**Operating system(s):** Unix/Linux

**Programming language:** C++

**License:** GPL-3

**Availability:** RNA-QC-Chain, including source code, documentation, and examples, is freely available for non-commercial use with no restrictions at http://bioinfo.single-cell.cn/rna-qc-chain.html or http://124.16.150.212/rna-qc-chain.html

## Abbreviations

HMM: hidden Markov model
HTS: high-throughput sequencing
NGS: next-generation sequencing
PE: paired-end
QC: quality control
RPKM: reads per kilobases per millionreads
rRNA: ribosomal RNA
SD: Sprague–Dawley
UCSC: the University of California, Santa Cruz
